# Effect of human very low-density lipoproteins on cardiotrophin-like cytokine factor 1 (CLCF1) activity

**DOI:** 10.1038/s41598-018-22400-y

**Published:** 2018-03-05

**Authors:** Sarah Pasquin, Salma Chehboun, Agnieszka Dejda, Yasmine Meliani, Virginia Savin, Gregory J. Warner, Roger Bosse, Aurélie Tormo, Gaétan Mayer, Mukut Sharma, Przemyslaw Sapieha, Catherine Martel, Jean-François Gauchat

**Affiliations:** 1 Département de pharmacologie et physiologie, Université de Montréal, Montreal, QC H3T 1J4 Canada; 2 Département de Biochimie et Médecine Moléculaire, Université de Montréal, Montreal, QC H3T 1J4 Canada; 3 Renal Division, KCVA Medical Center, Kansas City, MO 64128-2226 USA; 4 Perkin Elmer, 940 Winter Street, Waltham, MA 02451 USA; 5 Faculté de Pharmacie, Université de Montréal, Montreal, QC H3T 1J4 Canada; 6 Département de Médecine, Université de Montréal, Montreal, QC H3T 1J4 Canada

## Abstract

The cytokines CLCF1 and CNTF are ligands for the CNTF receptor and the apolipoprotein E (ApoE) receptor sortilin. Both share structural similarities with the N-terminal domain of ApoE, known to bind CNTF. We therefore evaluated whether ApoE or ApoE-containing lipoproteins interact with CLCF1 and regulate its activity. We observed that CLCF1 forms complexes with the three major isoforms of ApoE in co-immunoprecipitation and proximity assays. FPLC analysis of mouse and human sera mixed with CLCF1 revealed that CLCF1 co-purifies with plasma lipoproteins. Studies with sera from ApoE^−/−^ mice indicate that ApoE is not required for CLCF1-lipoprotein interactions. VLDL- and LDL-CLCF1 binding was confirmed using proximity and ligand blots assays. CLCF1-induced STAT3 phosphorylation was significantly reduced when the cytokine was complexed with VLDL. Physiological relevance of our findings was asserted in a mouse model of oxygen-induced retinopathy, where the beneficial anti-angiogenic properties of CLCF1 were abrogated when co-administrated with VLDL, indicating, that CLCF1 binds purified lipoproteins or lipoproteins in physiological fluids such as serum and behave as a “lipocytokine”. Albeit it is clear that lipoproteins modulate CLCF1 activity, it remains to be determined whether lipoprotein binding directly contributes to its neurotrophic function and its roles in metabolic regulation.

## Introduction

Cardiotrophin-like cytokine (CLCF1) is an IL-6 family cytokine^1,2^ efficiently secreted as a complex with the soluble cytokine receptor cytokine-like factor 1 (CRLF1)^3,4^. CLCF1 activates the tripartite ciliary neurotrophic factor receptor (CNTFR), comprised of CNTFRα, gp130 and LIFRβ^5^. CLCF1 and CNTF share signaling pathways^5,6^ suggesting that the extensive pre-clinical and clinical investigations conducted on CNTF might also be indicative of the therapeutic potential of CLCF1. CNTF demonstrated potent neurotrophic activities *in vitro* and in animal models of neurodegenerative diseases, leading to clinical trials in patients suffering from Huntington’s disease, amyotrophic lateral sclerosis and promising effects in retinal degeneration pathologies^7–11^. Systemic administration of CNTF resulted in unexpected substantial weight loss^8^. This observation triggered pre-clinical investigations of CNTF in models of metabolic syndrome. Daily CNTF injections led to long term anorexic effects in diet-induced obese and leptin-deficient mice^12,13^. CNTF decreased hyperglycemia and hyperinsulinemia suggesting that it could overcome leptin resistance in obese individuals and benefit type II diabetics^12,13^. The effects of CNTF are believed to be both central and systemic, as CNTFR is expressed in the region of the brain controlling energy balance, in adipose tissues and in skeletal muscle^12,14–16^.

Thanks to these promising preclinical results, a CNTF derivative was tested in clinical trials in which significant weight loss was observed^17,18^, indicating that activation of the CNTFR can regulate food intake and metabolism.

The existence of a second CNTFR ligand had been predicted from, i) dramatic differences between the phenotypes of CNTFR and CNTF deficient mice^19^; ii) observations that CNTF is a cytoplasmic cytokine devoid of signal peptide^20,21^; iii) identification of a CNTF-inactivating mutation homozygous in 2–3% of the study population, without observable neuropathology^22^. The role of CLCF1 as a key CNTFR ligand during development is corroborated by similarities in the phenotypes of CLCF1, CRLF1 and CNTFR deficient mice^19,23–25^ and the overlap between the syndromes associated with mutations in *CLCF1*, *CRLF1* and *LIFRβ* genes^4,26–28^. Mutations in *CRLF1* and *CLCF1* genes result in Crisponi (MIM 601378) and cold-induced sweating (CISS; MIM 272430 and 610313) syndromes, two rare overlapping recessive conditions^4,26,27,29^. Infants with these conditions manifest marked disinterest in food^4,26,27,29^ suggesting CLCF1, alike CNTF can regulate food intake. Crisponi syndrome patients tend to suffer from high grade fever episodes and most die during the first year of life^4,26^, supporting a role of CLCF1 in the regulation of metabolism.

Kidney cells express CNTFR^30^. CLCF1 and CRLF1 are detectable in the developing kidney and can induce the development of mature nephron structure *in vitro*^31^. A urinary system anomaly has been reported in a CISS patient with a mutation in CRLF1^32^, supporting a role of CLCF1 in kidney development. Our results^33^ suggest an involvement of CLCF1 in focal segmental glomerulosclerosis (FSGS), the most common primary glomerular cause of renal disease in children and adults^34^. Numerous lines of evidence support the presence of a circulating factor in FSGS patient with recurrence^33^. We identified CLCF1 in the serum of FSGS patients and observed that CLCF1 activates the STAT3 signaling in glomeruli and podocytes and induces albuminuria in mice and proteinuria in rats^33^, indicating that this cytokine could represent such factor.

Normal serum comprises components that can inactivate the permeability inducing factor detected in FSGS sera. Fractionation studies of sera from healthy volunteers identified ApoE as an anti-permeabilty factor^35^. This suggests CLCF1 could interact with ApoE. In support of this hypothesis, lipoprotein apheresis, a procedure that depletes ApoE complexed with lipids^36^, is used as a therapeutic option for steroid-resistant FSGS^37,38^ and CNTF was shown to interact with ApoE *in vitro*^39^.

Interestingly, recent studies have demonstrated an association between ApoE variants and aberrant lipid metabolism in age-related macular degeneration^40–43^. CNTF is a potent treatment in retinal degeneration pathologies, such as retinitis pigmentosa, underlying a possible interaction between CNTF and ApoE containing lipoproteins in the retina^11,44–46^.

We observed that CLCF1, like CNTF^39^, binds ApoE an important modulator of lipids transport and metabolism. CLCF1, when incubated with serum or purified lipoproteins, participates in lipoprotein-lipid complexes. Formation of complexes with lipoprotein particles changes the biological activity of CLCF1 on CNTFR transfectants and CNTFR expressing cell lines and tissues. This indicates that CLCF1 is likely a “lipocytokine” in serum or cerebrospinal fluid and, that lipoprotein may regulate the neurotrophic and metabolic functions of this cytokine as well as its pathological role in FSGS.

## Results

### CLCF1 forms complexes with ApoE2, 3 and 4

**Figure 1:**
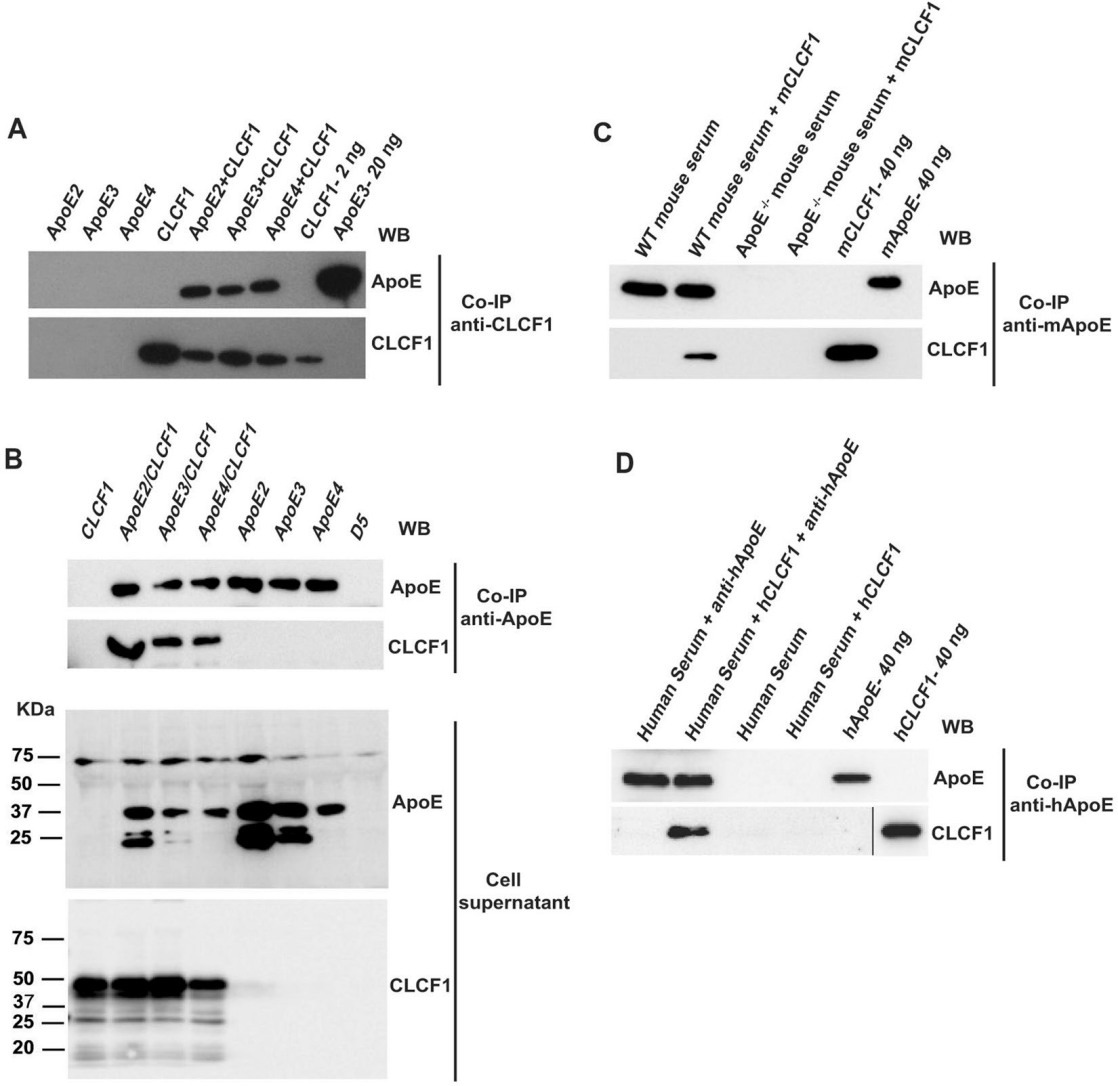
CLCF1 binds recombinant and serum ApoE. (**A**) Recombinant CLCF1 (200 ng) and ApoE2, 3 or 4 (250 ng) were incubated alone or in combination for 16 h. The samples were subjected to immunoprecipitation (IP) with anti-CLCF1 and protein G agarose. Immunoprecipitated proteins were analyzed by WB using mAbs specific for ApoE (upper panel) or CLCF1 (lower panel). Lanes “CLC 2 ng” and “ApoE3 20 ng”, show results of recombinant proteins directly subjected to SDS-PAGE and WB blot analysis. (**B**) CLCF1 and ApoE form a complex when co-expressed in HEK-293 cells. The cell culture medium from stable transfectants expressing the indicated proteins in combination with CRLF1 or from cells transfected with empty expression vector (lane D5) was subjected to immunoaffinity chromatography with rat-anti-human ApoE and anti-rat Ig Agarose. The eluates were analysed by WB using anti-ApoE or anti-CLC mAbs (upper panels). Supernatants of the stable transfectants were analyzed for the presence of ApoE or CLCF1 by WB (lower panels). (**C** and **D**) CLCF1 binds mouse and human serum ApoE. (**C**) Mouse serum samples isolated from WT or ApoE^−/−^ mice were diluted 1:20, mixed with biotinylated mouse CLCF1 and the complexes immunoprecipitated with rabbit anti-mouse ApoE and anti-rabbit Ig agarose. The eluates were analysed for ApoE or CLCF1 by WB using anti-mouse ApoE or HRP-labelled streptavidin. The last two lanes show the signals obtained with 40 ng of biotinylated mouse CLCF1 or ApoE included as control. (**D**) Human serum samples were diluted 1:20, mixed with biotinylated human CLCF1 and the complexes immunoprecipitated with rat anti-ApoE and anti-rat Ig agarose. The eluates were analyzed for ApoE or CLCF1 by WB using anti-ApoE or HRP-labelled streptavidin. The last two lanes show the signals obtained with 40 ng of biotinylated human CLCF1 or ApoE included as control. Blots’ images where cropped to show relevant areas.

As an initial test to determine whether CLCF1 formed a complex with ApoE, we incubated biotinylated CLCF1 with ApoE and immunoprecipitated ApoE using an anti-CLC mAb. As reported for CNTF^39^, co-immunoprecipitation of CLCF1 with the three most common ApoE isoforms, ApoE2, 3 and 4 could be observed (Fig. 1A). We next analyzed whether CLCF1 and ApoE form complexes when co-expressed in transfectants. Efficient secretion of CLCF1 by fibroblasts requires co-expression of the soluble cytokine receptor CRLF1^4,5^. We therefore generated stable tranfectants expressing ApoE2, 3 or 4, co-expressing CLCF1 and CRLF1 or co-expressing CLCF1 and CRLF1 with either ApoE2, 3 or 4. As expected, ApoE2, 3 and 4 or CLCF1 could be detected in the culture medium of the cells transfected with the corresponding cDNA (Fig. 1B lower panels). Isolation of ApoE2, 3 and 4 from the transfectants culture medium by affinity chromatography using anti-ApoE mAb resulted in co-isolation of CLCF1 (Fig. 1B upper panels). This confirmed the ApoE-CLCF1 interaction (Fig. 1A) when ApoE2, 3 or 4 was co-incubated with CLCF1 and also indicated that the formation of ApoE-CLCF1 complex was not prevented by CRLF1.

To avoid potential competition between exogenous or secreted ApoE and fetal bovine serum ApoE, these experiments were performed using buffered solutions (Fig. 1A) or serum-free cell culture medium (Fig. 1B). Serum ApoE is involved in transport of bound lipids. The formation of complexes with lipids is believed to strongly affect ApoE structure including the four helix NH_2_ domain that resembles type I cytokines^47,48^. We therefore determined whether CLCF1 could also form complexes with serum ApoE. We mixed mouse CLCF1 with serum isolated from WT or ApoE^−/−^ mice and subjected the mixture to immunoaffinity chromatography using anti-mouse ApoE mAb. Co-immunoprecipitation of CLCF1 and ApoE could be detected (Fig. 1C). However, similar immunoaffinity chromatography using a mixture of CLCF1 and ApoE deficient mouse serum did not result in co-immunoprecipitation thus ruling out a non-specific isolation of CLCF1 (Fig. 1C). As it is unclear whether mouse ApoE should be considered as ApoE3 or ApoE4 subtype^49,50^, we examined whether CLCF1 forms a complex with human serum ApoE3 (Fig. 1D). As observed with mouse serum, co-immunoprecipitation of human serum ApoE and CLCF1 was detected, indicating that formation of the CLCF1-ApoE complex could be detected in the presence of serum proteins and lipids (Fig. 1D).

### Complexes between ApoE2, 3 and 4 and CLCF1 can be detected in solution using an AlphaLISA proximity assay

**Figure 2:**
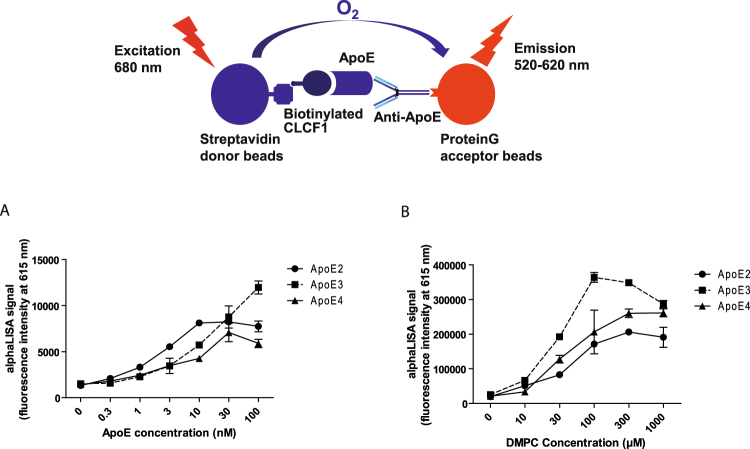
AlphaLISA proximity assay shows CLCF1-ApoE complex formation that is increased by the lipid DMPC. (**A**) ApoE2, 3 or 4 were mixed with CLCF1 (10 nM) in PBS 0.1% BSA for 1 h with IgG rat anti-ApoE (3 nM) and anti-IgG rat acceptor beads (10 μg/mL). Streptavidin donor beads (40 μg/mL) were further added for 1 h and 615 nm fluorescence signal assessed. (**B**) The indicated ApoE isotypes (30 nM) were mixed at 37 °C with increasing amount of DMPC for 1 h. ApoE-DMPC mix were further incubated with CLCF1 and subjected to AlpahLISA proximity assay. Histograms indicate the mean fluorescence intensity of 3 independent samples ± SD.

We next examined whether the formation of complexes could also be observed in solution using a proximity assay. In this assay biotinylated CLCF1 is used to recruit the streptavidin coated donor beads while the anti-rat IgG acceptor beads are recruited by the anti-ApoE mAb/ApoE, putative CLCF1 binding partner (Fig. 2). A strong energy transfer was detected between the donor and acceptor beads, indicating that interaction between CLCF1 and ApoE2, 3 and 4 could be detected in solution. (Fig. 2A). The observed signal was more that 10 × higher than the background energy transfer in the absence of CLCF1, ApoE or anti-ApoE mAb, indicating that it was induced by the formation of CLCF1-ApoE-anti-ApoE complexes (Fig. 2A).

### CLCF1-ApoE complexes are not disrupted by lipids

Having shown that CLCF1-ApoE can be detected in solution using the AlphaLISA proximity assay (Fig. 2A), we used this approach to assess the effect of lipid-induced change in ApoE structure on the interaction between CLCF1 and ApoE. While our data on co-immunoprecipitaiton between serum ApoE and CLCF1 suggest that ApoE can interact with CLCF1 in the presence of lipids, the formation of complex between lipid-free ApoE and the cytokine could not be excluded. 1,2-dimyristoyl-sn-glycero-3-phosphocholine (DMPC) is extensively used as a model lipid that binds and induces ApoE conformation changes^47^. We therefore investigated the effect of DMPC on CLCF1-ApoE3 interaction. Preincubation of ApoE with DMPC at concentration previously used to alter ApoE four α-helix NH_2_ domain structure^47^ markedly increased the proximity assay signal (Fig. 2B), indicating that lipid-bound ApoE, which is believed to represent the bulk of extracellular ApoE, can still form a complex with CLCF1. Interestingly, the interaction between the predominant ApoE isotype, ApoE3, and CLCF1 was more sensitive to the effect of DMPC than ApoE2-CLCF1 or ApoE4-CLCF1 according to the proximity assay signal (Fig. 2B).

### ApoE increases the biological activity of CLCF1

**Figure 3:**
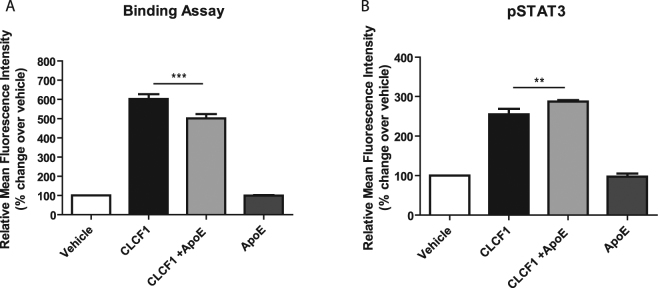
ApoE3 increases CLCF1-induced CNTFR activation. (**A**) Ba/F3 transfected with CNTFRα, gp130 and LIFRβ cDNA were successively incubated with mouse biotinylated CLCF1 (1 μg/ml) alone or in the presence of ApoE3 (10 mg/ml) and PE-labelled streptavidin and fluorescence was assessed by flow cytometry. Vehicle: cells incubated with PE-streptavidin alone. (**B**) Ba/F3-CNTFR were stimulated with CLCF1 (100 ng/ml) or ApoE3 (10 mg/ml) alone or in combination for 15 min. STAT3 tyrosine phosphorylation was assessed by intracellular staining with anti-pSTAT3 mAb and flow cytometry. Vehicle: cells incubated with PE-streptavidin alone. Histograms indicate the mean fluorescence intensity of 3 independent samples ± SD.

Next, we determined whether ApoE could modify CNTFR recruitment and activation using Ba/F3 transfectants expressing the tripartite CNTFR^5^. Interestingly, while the binding of biotinylated CLCF1 was slightly decreased in the presence of ApoE3 (Fig. 3A), CLCF1-induced STAT3 phosphorylation was increased in the presence of ApoE3 (Fig. 3B).

### CLCF1 co-purifies/co-elutes with ApoE-containing lipoprotein complexes

**Figure 4:**
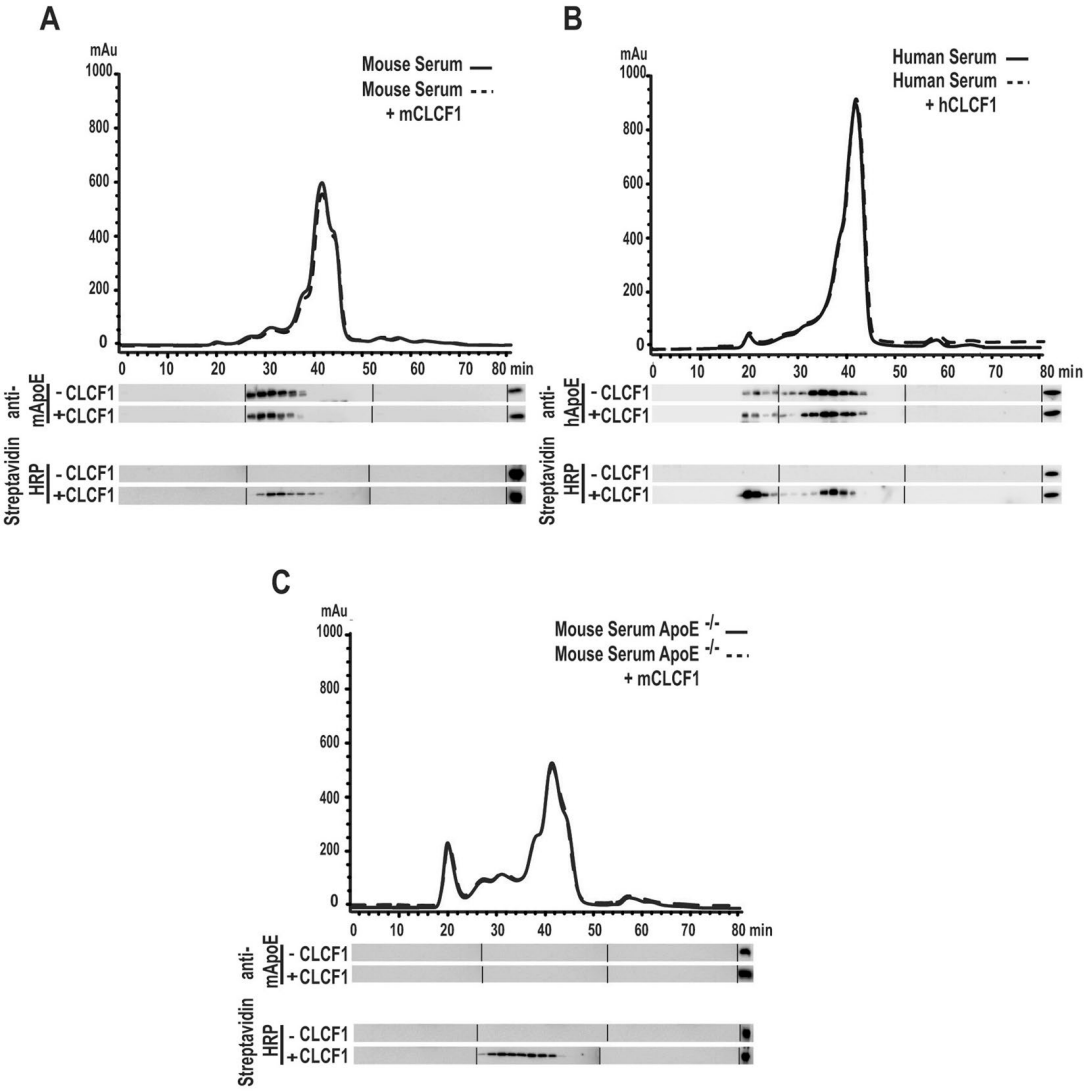
CLCF1 co-purifies or co-elutes with serum lipoprotein complexes. (**A**) WT mouse, (**B**) human or (**C**) ApoE^−/−^ mouse sera were mixed with corresponding biotinylated CLCF1. The mixtures were fractionated by FPLC using a Superose 6 HR 10/ column in order to obtain 40 fractions. The fractions were analyzed for ApoE or CLCF1 by WB using anti-ApoE or HRP-labelled streptavidin. The right lanes show the signals obtained with 40 ng of ApoE or biotinylated CLCF1 included as control. Blots’ images where cropped to show relevant areas.

This observation prompted us to examine whether CLCF1 participated in ApoE-containing lipoprotein complexes. We therefore subjected CLCF1 mixed with mouse or human serum to Fast Performance Liquid Chromatography (FPLC). This approach is widely used to analyze lipoprotein complexes^51^. CLCF1 was detected in the ApoE containing fractions suggesting that the two proteins form complexes (Fig. 4A,B). No CLCF1 could be revealed in the fractions containing free proteins or small size protein complexes, indicating that CLCF1 is mainly bound to large size, presumably lipoprotein complexes. This is further supported by the data obtained when CLCF1 was mixed with sera from ApoE^−/−^ mice. The lipoprotein elution profile is modified in these mice i.e. the proportion of VLDL, IDL and LDL is markedly increased while that of HDL is decreased^52^. Although the CLCF1 elution profile was altered (Fig. 4C), CLCF1 remained in lipoprotein fractions. This suggests that CLCF1 can interact with lipoprotein complexes independent of ApoE and that it represents a “lipocytokine”.

### CLCF1 binds VLDL and LDL

**Figure 5:**
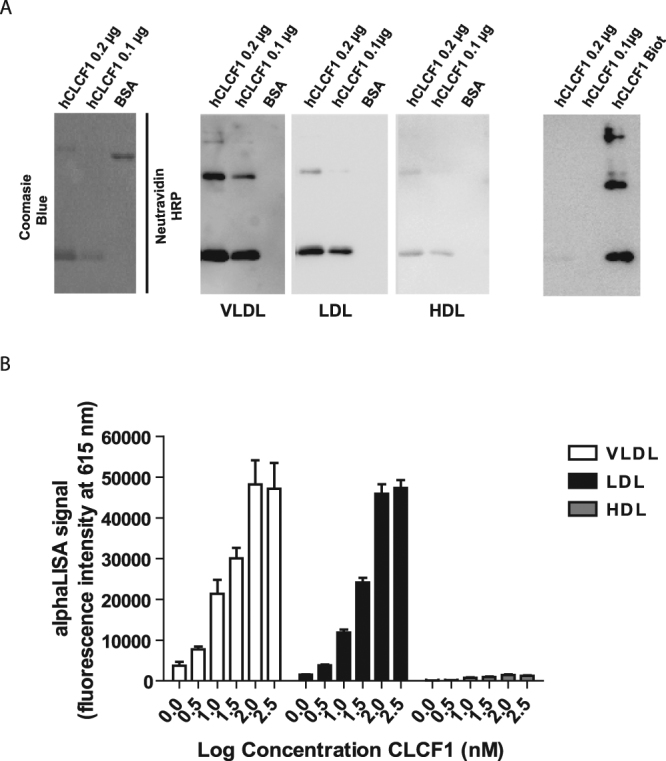
Biotinylated LDL and VLDL bind CLCF1. (**A**) Interaction between CLCF1 and lipoproteins was assessed using ligand blotting assays: 0.1–0.2 μg of hCLC, or 1 μg of BSA were subjected to non-reducing SDS-PAGE. Proteins were revealed by Coomassie blue staining (left section) or electro-transferred to nitrocellulose and incubated successively with 30 μg/ml biotinylated VLDL, LDL or HDL and HRP-labeled neutravidin (middle 3 panels). Signal was revealed by chemiluminescence. Image in the far right section shows results of incubation with HRP-labeled neutravidin alone. Lane hCLC biot; 40 ng of biotinylated CLC was used as positive control for neutravidin detection. Blots’ images where cropped to show relevant areas. (**B**) The formation of LDL and VLDL complexes can be detected using an AlphaLISA proximity assay. Biotinylated hCLCF1 was mixed with LDL, HDL and VLDL covalently coupled to acceptor beads (10 μg/mL). Streptavidin donor beads (40 μg/mL) were further added for 1 h and 615 nm fluorescence signal assessed. Histograms indicate the mean fluorescence intensity of 3 independent samples ± SD.

To test whether CLCF1 can bind lipoproteins we used a ligand blotting approach used to detect LDL receptors^53^. CLCF1 was subjected to SDS-PAGE under non-reducing conditions, transferred to nitrocellulose and incubated with biotinylated LDL, HDL or VLDL (Fig. 5A). A clear signal was detected when CLCF1 monomers and multimers were incubated with VLDL and LDL while no signal could be observed with BSA, used as a specificity control or when CLCF1 was incubated only with the reagent used to detect biotinylated lipoproteins (Fig. 5A left panel). A very faint signal was observed with HDL.

To confirm CLCF1-lipoprotein interactions, we performed AlphaLISA proximity assays between biotinylated CLCF1 (to recruit streptavidin coated donor beads) and VLDL, LDL or HDL covalently conjugated to acceptor beads. Strong fluorescent signals where obtained when CLCF1 was mixed with LDL and VLDL (Fig. 5B). As observed with the ligand blot assays, no clear positive signals were detected when the proximity assay was performed using HDL-conjugated beads (Fig. 5B). To assess the specificity of the signal, we carried out competition experiments by adding increasing amount of unlabeled CLCF1 to protein-protein interactions assays. As expected, the presence of unlabeled CLCF1 decreased biotinylated CLCF1-LDL and CLCF1-VLDL proximity assay signal indicating that the latter was specific (Supplementary Figure S1). No competition was observed for the very low CLCF1-HDL signal suggesting that it was nonspecific. These results indicate that CLCF1 can efficiently participate to form complexes with LDL and VLDL lipoproteins.

### Lipoproteins modify CLCF1 binding to CNTFR-expressing transfectants and cell lines

**Figure 6:**
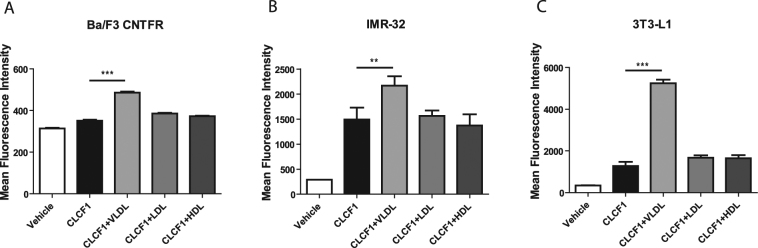
VLDL increases CLCF1 binding to CNTFR-expressing cells. Ba/F3 transfected with CNTFRα, gp130 and LIFRβ cDNA, IMR32 neuroblastoma or 3T3-L1 pre-adipocytes were successively incubated with species-matched biotinylated CLCF1 (1 μg/ml) alone or in the presence of LDL, HDL or VLDL (all at 100 μg/ml). Cells were then incubated with PE-conjugated streptavidin and fluorescence was assessed by flow cytometry. Vehicle: cells incubated with PE-streptavidin alone. Histograms indicate the mean fluorescence intensity of 3 independent samples ± SD.

Next, we investigated whether the formation of CLCF1-lipoprotein complex influences the binding of CLCF1 to Ba/F3 transfectants expressing the tripartite CNTFR (CNTFRα, gp130 and LIFRβ). When Ba/F3 cells were incubated with biotinylated CLCF1 in the presence of HDL or LDL, the binding of the cytokine was not significantly modified (Fig. 6A). Inclusion of VLDL markedly enhanced CLCF1 binding to the CNTFR transfectants (Fig. 6A). To exclude possibility that the VLDL-effect is linked to a characteristic of the Ba/F3 pro-B cell line, we investigated the effect of the lipoproteins on the binding of CLCF1 to IMR-32 human neuroblastoma cells or the 3T3-L1 mouse pre-adipocytes both expressing CNTFR^5,14^. As observed with the Ba/F3 CNTFR, the presence of VLDL strongly increases the binding to the CNTFR expressing cells (Fig. 6B and C). No CLCF1 binding could be detected on Ba/F3 expressing LIFRβ in the presence or absence of lipoproteins (data not shown).

### CLCF1 activity is modified by HDL, LDL and VLDL

**Figure 7:**
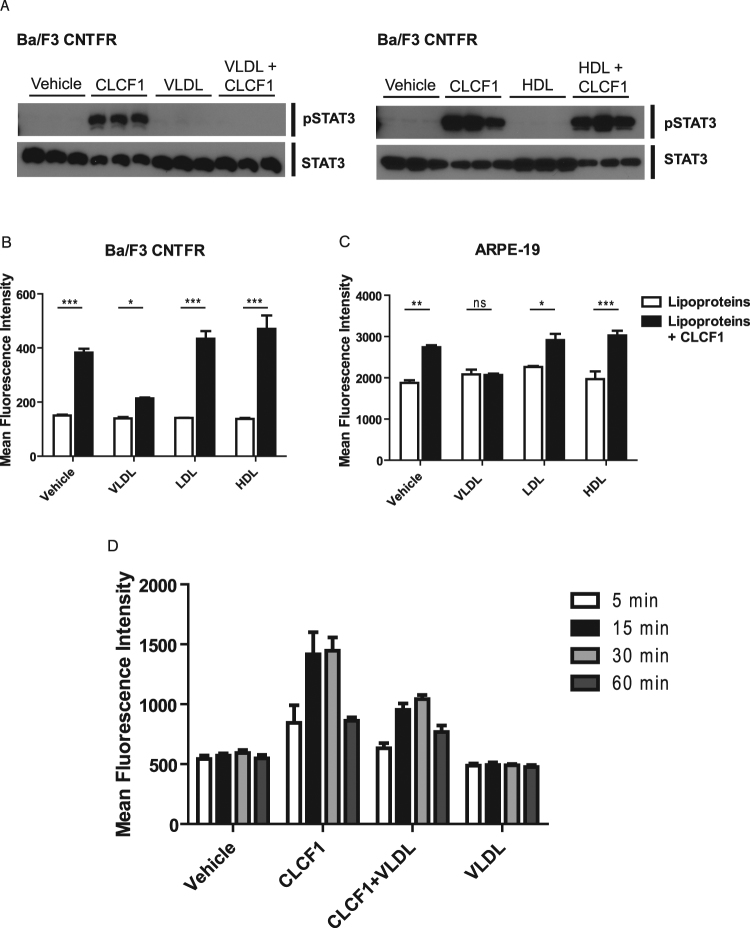
Lipoproteins modulate CLCF1-induced STAT3 tyrosine phosphorylation. (**A**,**B**) Ba/F3 transfected CNTFRα, gp130 and LIFRβ cDNA were stimulated with CLCF1 (100 ng/ml) or lipoproteins (100 μg/ml) alone or in combinations for 15 min. (**C**) ARPE-19 retinal epithelial cells were stimulated with CLCF1 (100 ng/ml) or lipoproteins (100 μg/ml) alone or in combinations for 5 min. (**D**) Ba/F3-CNTFR were stimulated with CLCF1 (100 ng/ml) or lipoproteins (100 mg/ml) alone or in combinations for 5–30 min. STAT3 tyrosine phosphorylation was assessed by Western Blot analysis (**A**) or by flow cytometry using intracellular staining with anti-pSTAT3 mAb (**B**–**D**). Vehicle: unstimulated cells. Histograms indicate the mean fluorescence intensity of 3 independent samples ± SD. Western blot analysis (**A**) is representative of 3 independent experiments. Blots were probed with antibodies specific for STAT3 or its tyrosine phosphorylated form (pSTAT3).

We next used CNTFR expressing Ba/F3 transfectants to evaluate whether binding to lipoprotein would modify the activity of CLCF1. We analyzed the activation of the CNTFR signaling pathways by phospho-specific flow cytometry^54^. CLCF1 did not induce a significant change of STAT3 phosphorylation in Ba/F3 cells when HDL or LDL concentration in the medium was close to serum levels^55^ (Fig. 7A). Inclusion of VLDL sharply reduced CLCF1-induced signaling (Fig. 7A). Time-course experiments indicated that the signaling reduction observed at 15 min in the presence of VLDL was not due to a delay in STAT3 activation (Fig. 7C).

In preclinical mouse models of retinopathy, activation of the JAK-STAT3 pathway by the CNTFR ligand CNTF was shown to be therapeutically beneficial in blocking pathological neovascularization and led to CNTF delivered by encapsulated cell intraocular implant as an established treatment for retinitis pigmentosa and macular telangiectasia^11,44–46^. We therefore tested the capacity of VLDL to inhibit CLCF1-induced STAT3 phosphorylation in ARPE-19 retinal epithelial cell line cultures. As observed with Ba/F3 transfectants, inclusion of VLDL markedly attenuated the CLCF1-induced phosphorylation of STAT3 (Fig. 7B). Altogether, our results indicate that VLDL, while increasing CLCF1 binding to its receptor, decreased the capacity of the CLCF1 receptor complexes to activate the JAK-STAT3 signaling pathway. Our results indicate that lipoproteins bind and regulate CLCF1 activity, suggesting that variation of the ratio between the different lipoproteins in serum or other body fluids could control the neurotrophic, metabolic and immunoregulatory functions of CLCF1.

### CLCF1 in oxygen induced retinopathy

**Figure 8:**
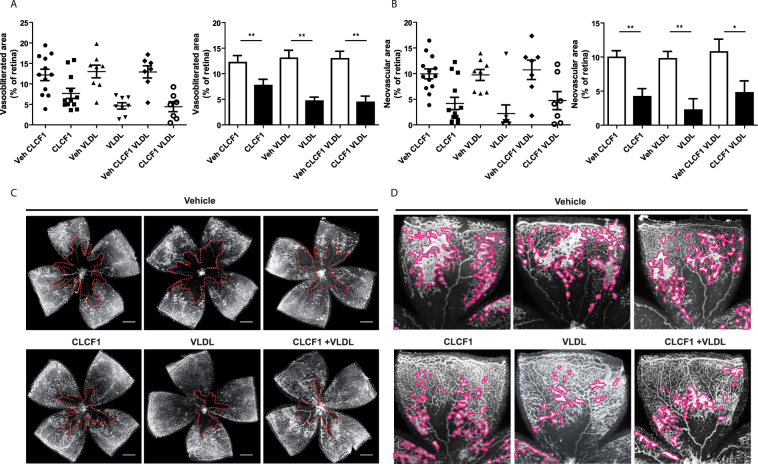
Treatment with CLCF1 or VLDL efficiently decreased oxygen-induced pathological neovascularization. C57BL/6 WT mice were subjected to OIR and injected intravitreally at P12 with CLCF1 (intravitral concentration 100 ng/mL), VLDL (intravitral concentration 10 μg/mL), a combination of CLCF1 and VLDL or vehicle. Results are expressed as percentage of (**A**) vaso-obliterated or (**B**) neovascular area versus the whole retinal area. Histograms bars represent mean value of percentage, and dots represent individual values ± SEM. (**C**) Representative retinal flatmount images are shown in with vaso-obliterated areas delimited with dotted line. (**D**) Representative retinal flatmount images are shown with pre-retinal neovascularization outlined. Scale bars: 500 µm.

In order the investigate the relevance of our findings *in vivo*, we interrogated on the ability of CLCF1 to inhibit angiogenesis in a mouse model of oxygen induced retinopathy that is widely used to study pathological neovascularization in the retina^56^. Mouse pups are placed in 75% O_2_ from postnatal day 7 to 12 to induce vascular degeneration and then returned to room air for 5 days. This model mimics retinopathy of prematurity and serves as a proxy for the angiogenic phase of proliferative diabetic retinopathy^57^. Intraocular injection of CLCF1 or VLDL individually significantly augmented vascular regeneration (Fig. 8A and C) and reduced pathological pre-retinal neovascularization (Fig. 8B and D) as determined at postnatal day 17. These findings are similar to those recently reported by Bucher and colleagues for CNTF alone^58^. Interestingly, injections of the CLCF1-VLDL complex did not result in additive beneficial effect compared with VLDL alone suggesting that VLDL prevents the effect of CLCF1 and, that the biological activity of CLCF1can be regulated by lipoproteins *in vivo* (Fig. 8).

## Discussion

CLCF1 is a potent neurotrophic factor with the ability to activate the tripartite CNTFR. Symptoms associated with mutations in the genes coding for CLCF1 or its binding partner CRLF1 suggest that this cytokine could regulate metabolism and food intake. In this study we observed that CLCF1, alike CNTF, forms complexes with ApoE, an important regulator of lipids transport and lipid metabolism. These complexes could be detected when the two binding partners were mixed or co-expressed using mouse or human proteins. CLCF1 also formed complexes with the three most common ApoE isotypes (ApoE2, 3 and 4). The formation of ApoE complexes was not prevented by co-expression with the soluble cytokine receptor CRLF1 that is necessary for efficient secretion of CLCF1 and recently shown to participate in CNTFR activation^59,60^. CLCF1-ApoE complexes could be observed using complementary approaches based on unrelated principles: co-immunoprecipitation, chromatography or proximity assays. The formation of ApoE-CLCF1 complexes was increased in presence of DMPC, a compound known to bind ApoE and previously used to investigate the lipid-induced changes of ApoE conformation^47^. This together with the observation that CLCF1 interacts with ApoE in lipid-containing serum indicates that CLCF1 is likely to bind ApoE in circulation. CLCF1-ApoE complexes could also be present in the central nervous system where ApoE plays a key role in lipid transport and regulation of neural function. CLCF1 has neuroprotective role during development in mouse models^24^. Interestingly, considering the high physiological concentration of ApoE in serum and cerebrospinal fluids, CLCF1 is expected to play its exocrine functions in an ApoE-complexed form. In support of this notion, no CLCF1 could be detected in the small protein fraction of mouse and human serum. While ApoE binds CLCF1, fractionation of ApoE-deficient mouse sera mixed with this cytokine indicates that ApoE is not required for the interaction of CLCF1 with lipoproteins. Further analysis revealed that CLCF1 binds with different plasma lipoproteins (LDL and VLDL). Thus, these results provide a strong rationale to consider CLCF1 as a “lipocytokine” and to pursue the investigation of its functions and pharmacology in lipid-complexed forms.

In line with its capacity to bind lipoprotein, CLCF1 has been shown to bind with high affinity to two lipoprotein receptors, sortilin and the sortilin-related multi-ligand receptor sorLA^59,60^. It will be of interest to evaluate whether CLCF1-lipoportein complexes mediate specific functions through this receptors and whether lipoproteins modify CLCF1 pharmacology by targeting the cytokine to cells expressing sortilin, sorLA or other lipoprotein receptors.

CLCF1 is a regulator of kidney development, of glomerular function and is believed to be a permeability factor implicated in the development and recurrence of the progressive renal disease FSGS^33^. Interestingly, depletion of lipoproteins by plasmapheresis is successfully used in treating FSGS and preventing kidney graft loss^59,60^. Therefore, we suggest, that CLCF1 could be the critical factor that is removed from the patients’ circulation during plasmapheresis. Future investigation on the regulatory role of ApoE and lipoproteins on the glomerular effect of CLCF1 might open new therapeutic approaches to treat FSGS.

We investigated the effect of ApoE and lipoproteins on CLCF1 biological activity through CNTFR. These experiments were significantly complicated by the fact that most biological assays are carried out in the presence of serum that contain lipoproteins. We found that addition of lipoproteins in proliferation assays strongly modified the background response even when we used delipidated serum or serum-free media, and prevented interpretation of the results (data not shown). Therefore, our experiments focused on signalling assays that could be performed in serum-free solutions and condition in which lipoprotein alone had no effect on the signalling cascade investigated. Data obtained regarding STAT3 phosphorylation, a key CNTFR signalling molecule activated by CLCF1^5,6^ indicate a significant modulation of the biological activity of CLCF1 by lipoproteins. Similar results were obtained with CNTFR transfectants and cell lines constitutively expressing CNTFR. Intriguingly, HDL-CLCF1 and LDL-CLCF1 complexes induced a slight but not significant increase of STAT3 phosphorylation *in vitro* compared to CLCF1 alone. The most striking effects were observed with VLDL: addition of this type of lipoprotein strongly reduced the induction of STAT3 phosphorylation by CLCF1. As VLDL did not inhibit CLCF1-CNTFR binding, this suggests that the binding of CLCF1 on VLDL interferes with the recruitment or the heterodimerization of the signaling chain LIFRβ and gp130. This prompted us to study the effect of VLDL in an *in vivo* assay in which activation of the CNTFR might be relevant for preclinical studies. Intraocular injection of either VLDL or CLCF1 in a mouse model of oxygen-induced retinopathy showed a beneficial effect on both retinal neovascularization and vaso-obliteration. No differences could be detected between VLDL alone and CLCF1 plus VLDL suggesting that the effect of CLCF1 is prevented by VLDL *per se*. Altogether, these results suggest that the cholesterolemic profile, (i.e. the concentration and the ratio between the different lipoproteins) could directly influence the biological activities of CLCF1 in functions such as neuron survival, immune response and metabolism. It would be of interest to investigate whether a disruption or an imbalance of CLCF1 activity regulation by lipoproteins could contribute to the deleterious effects observed in FSGS patients and whether recombinant ApoE or synthetic lipoprotein complexes could be beneficial in this pathology.

While our study focused on CLCF1, this cytokine shares biological activities and biochemical properties with other cytokines that may also interact with lipoproteins. Our results provide novel insight on lipoproteins and type 1 cytokine interactions and suggest that apolipoprotein- or lipoprotein-cytokine complexes could be used to modify the therapeutic properties of recombinant cytokines.

## Methods

### Production and purification of recombinant CLCF1

Mouse or human codon optimized synthetic cDNA (GeneArt™, Thermo Fisher Scientific Inc, Burlington, ON) coding for mature CLCF1 tagged with 6 his and a biotinylated Avitag^61^ or 6 his and a Flag (YKDDDDK) epitope were cloned in the expression vector pET24d (Novagen, Millipore Canada Ltd, Etobicoke, ON). The constructs were co-transformed with the BirA biotin-ligase coding vector pBirAcm (Avidity, Aurora, CO) in BL21 (DE3) (ThermoFisher Scientific). Expression was induced as described previously^62^ in THY medium supplemented with 12 mg/L biotin, 50 μg/ml kanamycin and 12.5 μg/ml chloramphenicol. Proteins were purified from the bacterial inclusion bodies by immobilized metal affinity chromatography^63^. Purifications were performed according to the instructions of the manufacturer (Qiagen Inc, Toronto, ON) using LPS free reagents and detoxified glassware. The recombinant proteins were renatured by dialysis against decreasing concentrations of urea, 100 mM glycine pH 3.5, 1 mM reduced glutathione, 0.1 mM oxidized glutathione followed by 1 mM reduced glutathione, 0.1 mM oxidized glutathione in 4 mM HCl and lastly 4 mM HCl. Purified cytokine was detoxified using polymyxin B-agarose (Sigma-Aldrich, Oakville, ON) to endotoxin levels below 0.1 EU/μg protein, assessed using a Limulus endotoxin assay (Pierce, ThermoFisher Scientific). Biological activity was measured using Ba/F3 transfected with the cDNA coding for mouse or human gp130, LIFRβ and CNTFRα and a Alamar Blue proliferation assay^64^.

### Generation of transfectants co-expressing CLCF1 and ApoE

Synthetic cDNA coding for hCLCF1/hCLRF1, ApoE2, 3 and 4 or the combinations of hCLCF1/hCLRF1 modified by the insertion of sequences coding for T2A “self-cleaving peptides”^65^ and the three ApoE isoforms were generated using synthetic cDNA (GeneArt™) and standard molecular biology methods^66^. The mono or multicistronic cDNA were inserted in the expression vector pcDNA™5/FRT vector and stable Flp-In™−293 cells transfectants generated and maintained according the Flp-In™ instruction manual (ThermoFisher Scientific).

### Analysis of CLCF1/ApoE interaction by co-immunoprecipitation

For co-immunoprecipitation assays between recombinant proteins, CLCF1 (200 ng) and ApoE 2, 3 or 4 (250 ng; BioVision, Cedarlane, Burlington, ON) were incubated alone or in combination for 16 h in 1 ml Opti-MEM™ (Life Technologies). CLCF1 was immunoprecipitated with 1 μg anti-CLCF1 mAb (R&D systems, Cedarlane, Burlington, ON) and 30 μl protein G agarose (Santa Cruz Biotechnology Inc, Dallas, TX). Co-immunprecipitated proteins were analyzed by Western Blotting (WB) using anti-CLCF1 or anti-ApoE mAbs (R&D systems, Cedarlane) and horseradish peroxidase (HRP)-labelled secondary Abs.

To analyze CLCF1-ApoE interaction in culture medium from Flp-In™ HEK-293 transfectants, cells stably transfected with pcDNA5 containing synthetic cDNAs coding for CLCF1/CRLF1, ApoE isotypes, combinations of these proteins or empty pcDNA™5/FRT were expanded to near confluence in DMEM supplemented with fetal calf serum (FCS) and then maintained for 5 days in Opti-MEM™ medium. Culture medium was concentrated 20 × by ultrafiltration centrifugation and analyzed for CLCF1 or ApoE by WB as described above. One ml concentrated culture medium aliquots were incubated with 1 μg rat anti-ApoE mAb (R&D systems) and 50 μl anti-rat Ig agarose resin (Sigma-Aldrich). Resin was washed with PBS and elution induced with 70 μl of 100 μM glycine pH 2.5 (glycine buffer). Neutralized eluates were analyzed by WB.

To investigate whether CLCF1 can form a complex with mouse serum ApoE, 30 μl aliquots of WT or ApoE^−/−^ mouse serum diluted 1:20 with PBS were incubated with or without 0.3 μg biotinylated mouse CLCF1 and 2.5 μg rabbit anti-mouse ApoE IgG for 16 h at 4 °C. Complexes were immunoprecipitated by adding 50 μl anti-rabbit Ig TrueBlot™ beads (Rockland Immunochemicals Inc, Limerick, PA). Beads were washed with 10 vol. PBS and retained proteins eluted with glycine buffer. Eluates were analyzed by WB using HRP-streptavidin (GE-Healthcare Life Science, Cedarlane, Burlington, ON) or rabbit anti-mouse ApoE Ab (Abcam Inc, Cedarlane) followed by HRP-labelled TrueBlot™ anti-rabbit Ab (Rockland Immunochemicals Inc).

To assess whether CLCF1 can form a complex with human serum ApoE, 50 μl aliquots human serum diluted 1:20 with PBS were incubated with or without 0.5 μg biotinylated CLCF1 and 2.5 μg rat-anti ApoE IgG for 16 h at 4 °C. Complexes immunoprecipitated by adding 50 μl anti-rat Ig agarose resin were washed with 10 vol. PBS. Glycine buffer eluates were analyzed by WB using HRP-streptavidin or rat-anti-ApoE and HRP-labelled anti-rat Ig.

### Analysis of ApoE/CLCF1 complexes by fast protein liquid chromatography (FPLC)

Aliquots of human, WT mouse or ApoE^−/−^ mouse serum (50 μl) were mixed with 1 μg biotinylated human or mouse CLCF1 and fractionated by FPLC using on Superose 6™ HR 10/30 column at the Lipid and Lipid Metabolite Analysis Core Facility (University of Alberta, Edmonton, AB). The 40 FPLC fractions were analyzed for ApoE by WB using anti-mouse ApoE IgG as described above or biotinylated anti-human ApoE IgG (Abcam Inc) and HRP-labelled streptavidin. CLCF1 was detected using HRP-labelled streptavidin.

### Analysis of ApoE-CLCF1 interaction by AlphaLISA

AlphaLISA protein-protein interaction assay was developed to evaluate CLCF1-ApoE interaction using streptavidin coated donor beads and anti-rat IgG acceptor beads (Perkin Elmer, Woodbridge, ON). ApoE2, 3 or 4 (0–100 nM) were mixed with CLCF1 (10 nM) in PBS 0.1% BSA for 1 h with rat IgG anti-ApoE (3 nM) (R&D systems, Cedarlane) and anti-rat IgG acceptor beads (10 μg/mL). Streptavidin donor beads (40 μg/mL) were further added for 1 h protected from light. Fluorescence was quantified using an Envision multilabel plate reader (Perkin Elmer). The signal without rat IgG anti-ApoE (background) was subtracted from the data.

The effect of lipid on CLCF1-ApoE interaction was assessed by AlphaLISA protein-protein interaction assay in presence of 1,2-dimyristoyl-*sn*-glycero-3-phosphocholine (DMPC) (0–1000 μM). ApoE2, 3, 4 (at 30 nM) were preincubated with increasing concentrations of DMPC for 1 h at 37 °C. ApoE-DMPC mix were further incubated with CLCF1 (at 10 nM) in PBS 0.1% BSA with rat IgG anti-ApoE (3 nM) and anti-rat IgG acceptor beads (10 μg/mL).Subsequently, streptavidin donor beads (40 μg/mL) were added and incubated for 1 h protected from light. Fluorescence was quantified as described above.

### Lipoprotein biotinylation

Lipoproteins (HDL, VLDL, LDL, 500 μg aliquots; Alpha Aesar, Ward Hill, MA) were biotinylated using a Sulfo-NHS-Biotin Kit (Pierce, ThermoFisher Scientific) for 1 h at 37 °C. Biotinylated lipoproteins were separated from free biotin by gel filtration on Sephacryl S-100 (GE Healthcare). Biotinylation was assessed by WB. Lipoproteins were diluted in 50 mM Tris-HCL pH 8, 2 mM CaCl_2_, 90 mM NaCl, 50 mg/ml BSA to a final concentration of 50 µg/ml for ligand blot experiments.

### Ligand Blot experiments with biotinylated lipoproteins and immobilized CLCF1

Aliquots of recombinant human CLCF1 (100–200 ng) were subjected to SDS-PAGE and electro-transferred to nitrocellulose membrane. The membranes were blocked in 50 mM Tris-HCL pH 8, 2 mM CaCl_2_, 90 mM NaCl, 50 mg/ml BSA and incubated with biotinylated lipoproteins at 50 μg/ml for 1 h. Membrane-bound lipoproteins were revealed using HRP-labelled neutravidin (ThermoFisher Scientific). One μg aliquots of LDLR, VLDLR, and SR-B1 (all from Alpha Aesar) were used as positive controls.

### Analysis of lipoproteins-CLCF1 interaction by AlphaLISA

Lipoproteins-CLCF1 interactions were assessed by AlphaLISA proximity assay using streptavidin-coated donor beads and lipoproteins-conjugated AlphaLISA acceptor beads. For lipoproteins conjugations on acceptor beads, 500 μg of unconjugated acceptor beads (Perkin Elmer) where washed in PBS and centrifuged at 16 000 g for 15 min. The conjugation was performed by mixing the acceptor beads with 50 μg of LDL, HDL or VLDL in 200 μl of 130 mM phosphate buffer pH 8.0 containing 20 mM of NaBH_3_CN. Beads where incubated for 18 h at 37 °C, washed twice in 200 μl of 100 mM Tris-HCl pH 8.0 and stored at 5 mg/mL in 100 mM Tris-HCl 0,01% sodium azide.

For the interaction assays, biotinylated CLCF1 (10 nM) was mixed in PBS 0.1% BSA for 1 h with LDL, HDL or VLDL-conjugated acceptor beads (10 μg/mL). Streptavidin donor beads (40 μg/mL) were further added for 1 h protected from light. Fluorescence was quantified using an Envision multilabel plate reader. The signal without CLCF1 (background) was subtracted from the data. The specificity of the signal was assessed by including increasing concentrations of unlabeled CLCF1 (0–1000 nM) in the interaction assay.

### Quantification of CLCF1-induced STAT3 tyrosine phosphorylation

CLCF1 (100 ng/ml) was preincubated for 1 h at 37 °C with ApoE2, 3 or 4 (10 μg/ml) or purified HDL, LDL and VLDL (100 μg/ml; Alpha Aesar). Ba/F3 transfectants cells were starved for 4 h and stimulated for 15 min at 37 °C with CLCF1, ApoE (10 μg/ml), purified lipoprotein complexes (100 μg/ml) or combinations of these reagents in PBS. ARPE-19 retinal pigmented epithelial cells (American Type Culture Collection, Manassas, VA) were starved for 24 h, detached and stimulated for 5 min at 37 °C with CLCF1, purified lipoprotein complexes (100 μg/ml) or combinations of these reagents in PBS. For Western blot analysis, lysates of Ba/F3 transfectants were subjected to SDS-PAGE on 7.5% polyacrylamide gels and protein were electro-transferred to PVDF membranes. Total and phopho-STAT3 (Tyr 705) were detected using specific rabbit IgG (Cell signaling Technologies, Danvers, MA) and HRP-labelled anti-rabbit antibody (R&D systems, Cedarlane). For flow cytometry experiments, cells were fixed, permeabilized and stained with anti-phospho STAT3 Abs (from BD Biosciences, Mississauga, ON or eBioscience, Affimetrix, Cedarlane) as described previously^64,67^. Fluorescence was quantified with a FACSCanto II flow cytometer (BD Biosciences).

### CLCF1-CNTFR binding assays

CLCF1 (1 μg/ml) was preincubated for 1 h at 37 °C with ApoE2, 3 or 4 (10 μg/ml) or purified HDL, LDL and VLDL (100 μg/ml). Ba/F3 tranfectants, IMR32 neuroblastoma cells, or 3T3-L1 fibroblasts were incubated for 1 h on ice in PBS 0.1% BSA alone or supplemented with biotinylated CLCF1 (1 μg/ml), ApoE (10 μg/ml), lipoproteins complexes (100 μg/ml) or combinations of these reagents. CLCF1 binding was revealed using PE-labelled streptavidin (1 μg/ml) and flow cytometry^68^.

### Oxygen Induced Retinopathy

All procedures conformed to the Canadian Council on Animal Care guidelines and were approved by the Animal Ethics Committee of the Maisonneuve-Rosemont Hospital Research Centre. C57BL/6 mice were purchased from The Jackson Laboratory, Bar Arbor, ME. Mouse pups and their fostering mothers (CD1, Charles River) were exposed to 75% O_2_ from P7 to P12 and returned to room air^57^. This model serves as a proxy to human ocular neovascular diseases such as diabetic retinopathy, which is characterized by a late phase of destructive pathological angiogenesis^69,70^. Mice were injected intraoccularly at P12 with CLCF1 (final intraocular concentration: 100 ng/mL), VLDL (10 μg/mL) or a combination of both. The retinas were dissected at P17, flatten mounted and incubated overnight with fluoresceinated isolectin B4 (1:100) in 1 mM CaCl_2_ to determine extent of avascular area or neovascularization area using ImageJ and the SWIFT-neovascularization method^71^.

## Electronic supplementary material

Supplementary Figure 1
