# Supplementary material for: Effect of human very low-density lipoproteins on cardiotrophin-like cytokine factor 1 (CLCF1) activity
Source: Sci Rep. 2018 Mar 5;8:3990. doi: 10.1038/s41598-018-22400-y (PMC5838168; doi:10.1038/s41598-018-22400-y)
Supplement: Supplementary file 1 — Supplementary Figure 1 [file 41598_2018_22400_MOESM1_ESM.pdf]

Supplementary Information

Effect of human very low-density lipoproteins on cardiotrophin-like cytokine factor 1 (CLCF1) activity

Sarah Pasquin [1,†], Salma Chehboun[1,†], Agnieszka Dejda[2], Yasmine Meliani[1], Virginia Savin[3], Gregory, J. Warner[4], Roger Bosse[4], Aurélie Tormo[1], Gaétan Mayer[5], Mukut Sharma[3], Przemyslaw Sapieha[2], Catherine Martel[6] and Jean-François Gauchat[1, *]

[1] Département de Pharmacologie et physiologie, Université de Montréal, Montreal, QC, H3T 1J4, Canada

[2] Département de Biochimie et Médecine Moléculaire, Université de Montréal, Montreal, QC, H3T 1J4, Canada

[3] Renal Division, KCVA Medical Center, Kansas City, MO 64128-2226, USA

[4] Perkin Elmer, 940 Winter Street, Waltham, MA 02451, USA

[5] Faculté de Pharmacie, Université de Montréal, QC, H3T 1J4, Canada ¶

[6] Département de Médecine, Université de Montréal, Montreal, QC, H3T 1J4, Canada

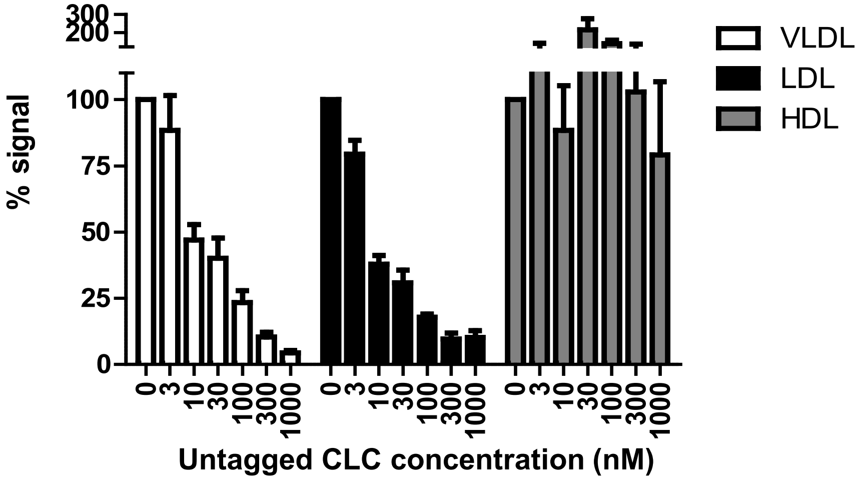

Figure S1. VLDL-CLCF1 and LDL-CLCF1 proximity assay signal can be competed-out using excess of non-biotinylated CLCF1. Biotinylated hCLCF1 was mixed with LDL, HDL and VLDL covalently coupled to acceptor beads (10 µg/mL) in presence of increased concentrations of unlabelled hCLC (as indicated in the figure). Streptavidin donor beads (40 µg/mL) were further added for 1 h and 615 nm fluorescence signal assessed. Histograms indicate the mean fluorescence intensity of 3 independent samples ± SD.

Figure 1-B

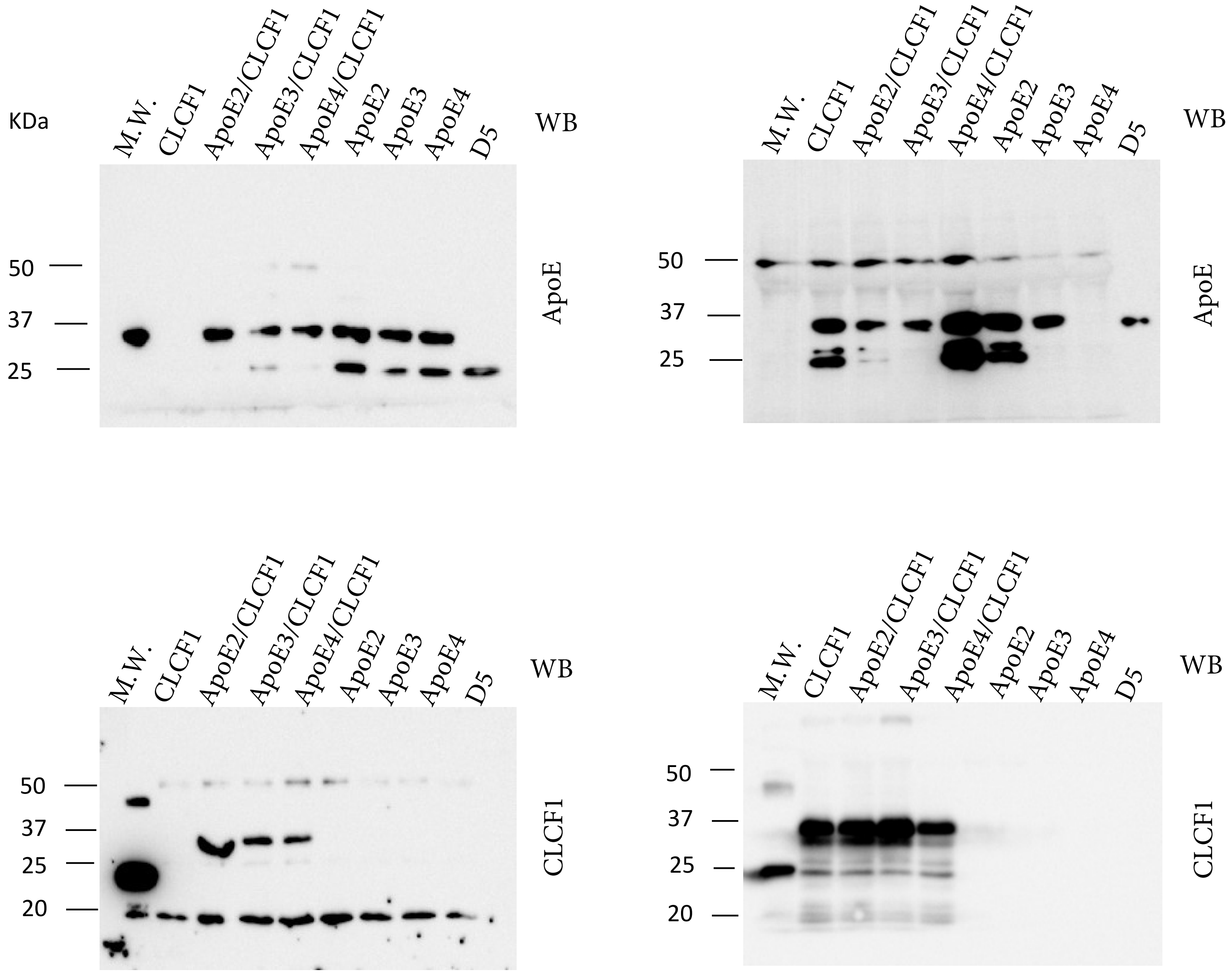
KDa
M.W.
CLCF1
ApoE2/CLCF1
ApoE3/CLCF1
ApoE4/CLCF1
ApoE2
ApoE3
ApoE4
D5
WB
50
37
25
ApoE
M.W.
CLCF1
ApoE2/CLCF1
ApoE3/CLCF1
ApoE4/CLCF1
ApoE2
ApoE3
ApoE4
D5
WB
50
37
25
ApoE
M.W.
CLCF1
ApoE2/CLCF1
ApoE3/CLCF1
ApoE4/CLCF1
ApoE2
ApoE3
ApoE4
D5
WB
50
37
25
20
CLCF1
M.W.
CLCF1
ApoE2/CLCF1
ApoE3/CLCF1
ApoE4/CLCF1
ApoE2
ApoE3
ApoE4
D5
WB
50
37
25
20
CLCF1

Figure 1-C

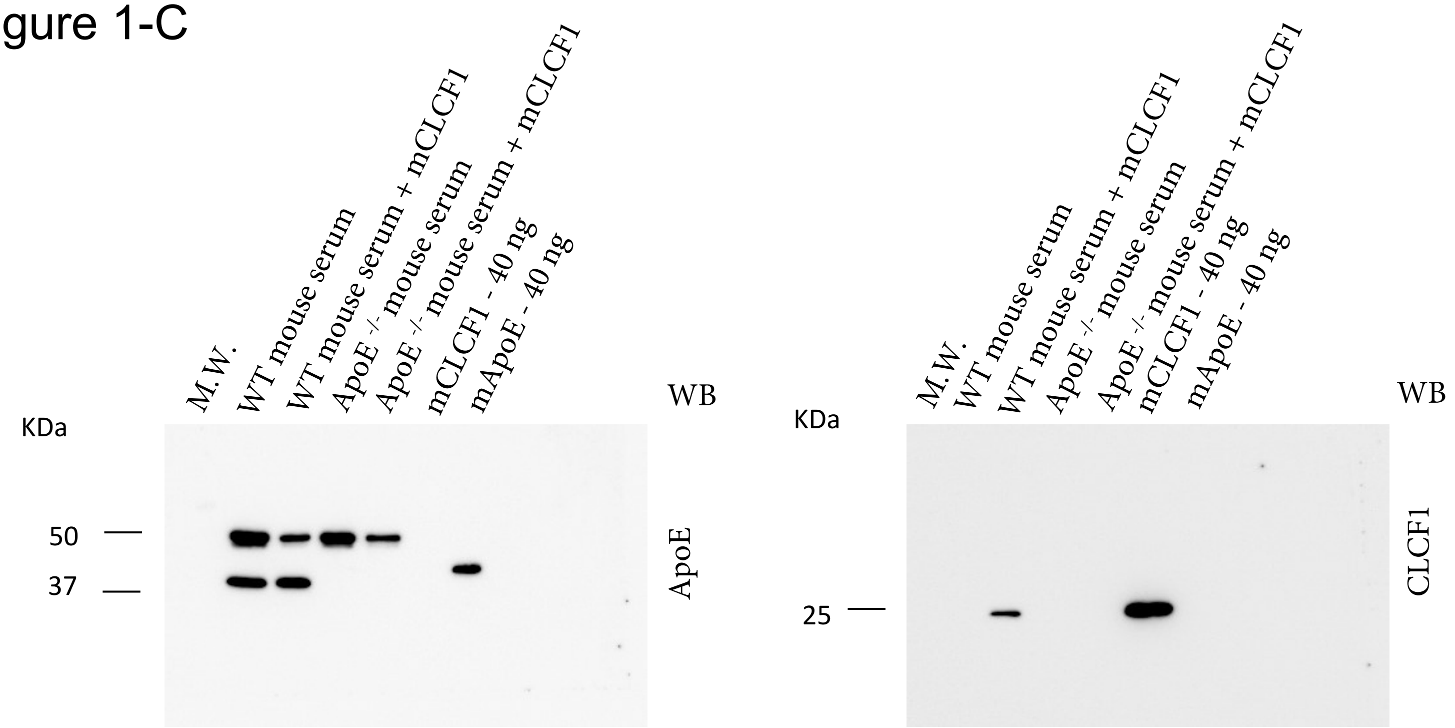

Figure 1-D

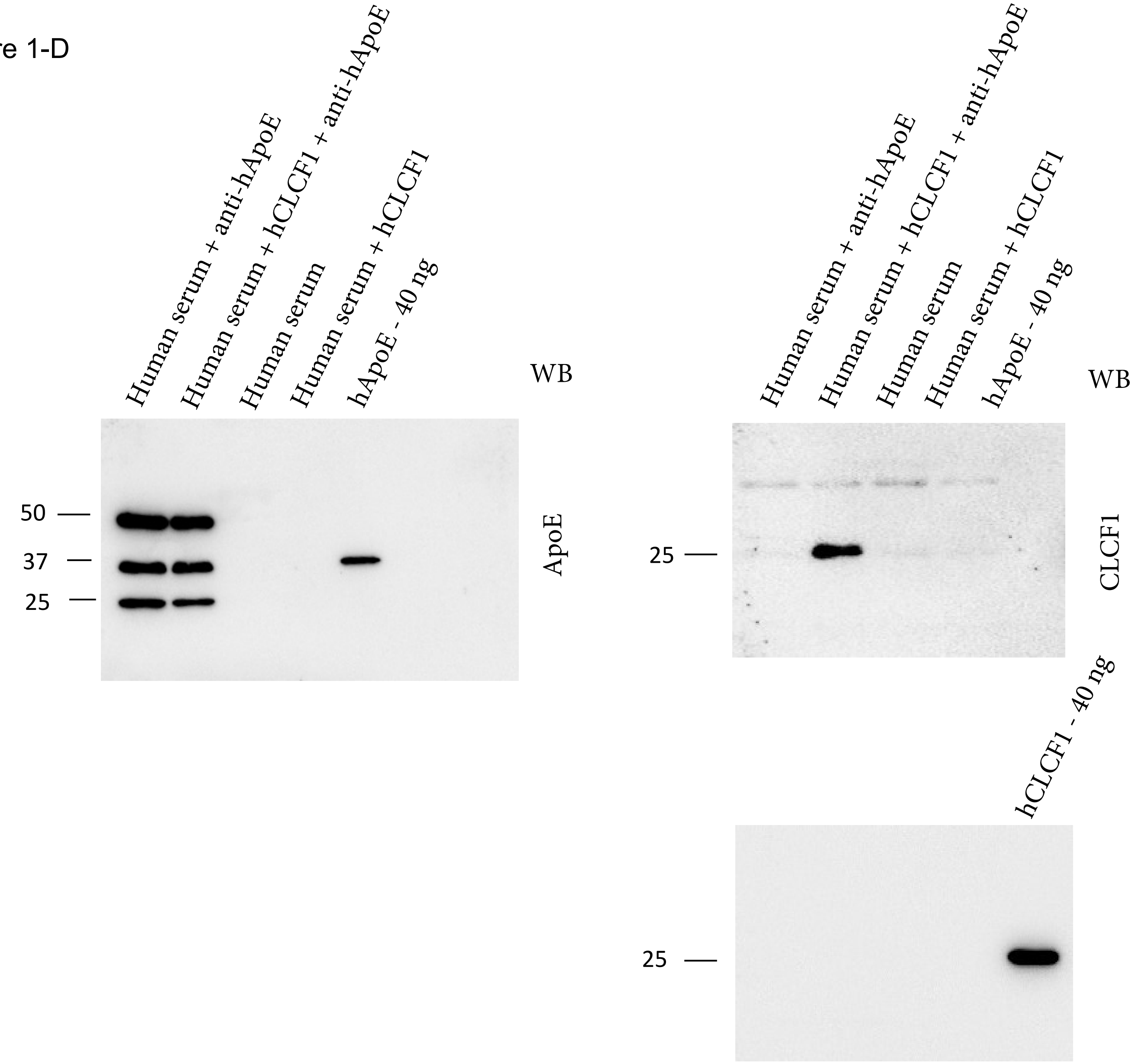

Figure 4-A

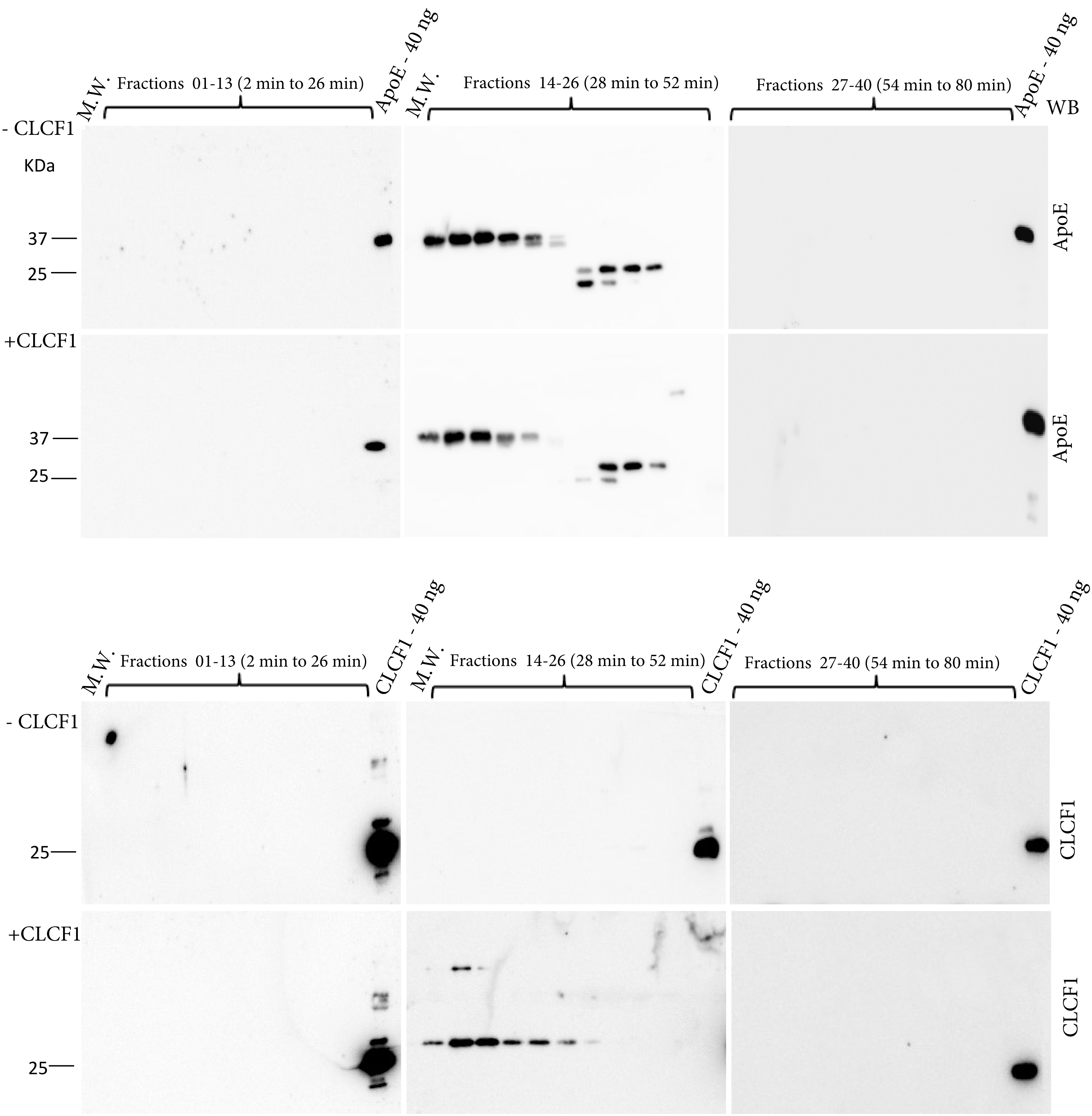
M.W.
Fractions 01-13 (2 min to 26 min)
ApoE - 40 ng
M.W.
Fractions 14-26 (28 min to 52 min)
Fractions 27-40 (54 min to 80 min)
ApoE - 40 ng
WB
- CLCF1
KDa
37
25
ApoE
+CLCF1
37
25
ApoE
M.W.
Fractions 01-13 (2 min to 26 min)
CLCF1 - 40 ng
M.W.
Fractions 14-26 (28 min to 52 min)
CLCF1 - 40 ng
Fractions 27-40 (54 min to 80 min)
CLCF1 - 40 ng
- CLCF1
25
CLCF1
+CLCF1
25
CLCF1

Figure 4-B

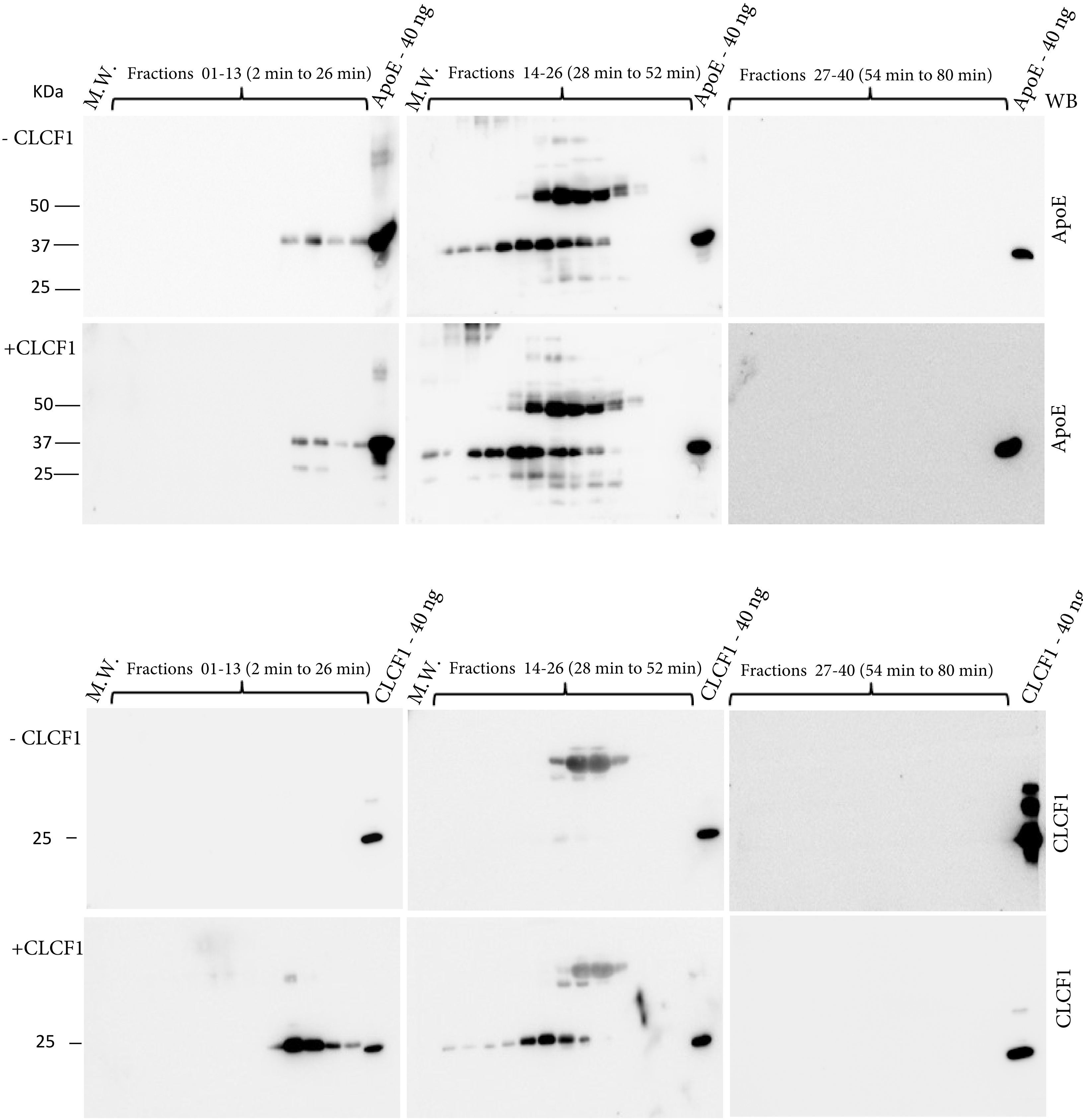
KDa
M.W.
Fractions 01-13 (2 min to 26 min)
ApoE - 40 ng
M.W.
Fractions 14-26 (28 min to 52 min)
ApoE - 40 ng
Fractions 27-40 (54 min to 80 min)
ApoE - 40 ng
WB
- CLCF1
50
37
25
ApoE
+CLCF1
50
37
25
ApoE
M.W.
Fractions 01-13 (2 min to 26 min)
CLCF1 - 40 ng
M.W.
Fractions 14-26 (28 min to 52 min)
CLCF1 - 40 ng
Fractions 27-40 (54 min to 80 min)
CLCF1 - 40 ng
- CLCF1
25
CLCF1
+CLCF1
25
CLCF1

Figure 4-C

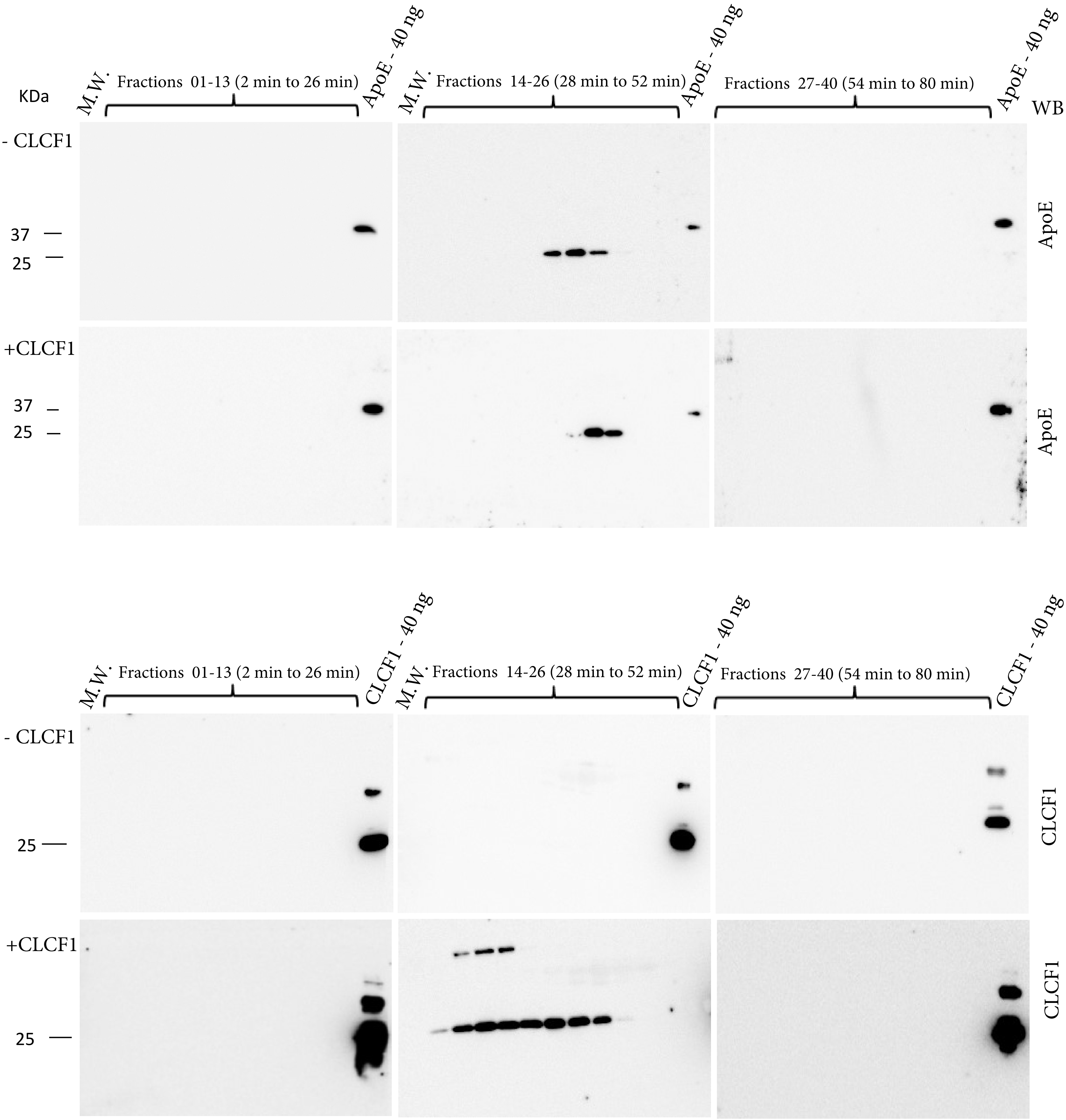
KDa
M.W.
Fractions 01-13 (2 min to 26 min)
ApoE - 40 ng
Fractions 14-26 (28 min to 52 min)
Fractions 27-40 (54 min to 80 min)
WB
- CLCF1
+CLCF1
37
25
ApoE
CLCF1 - 40 ng
CLCF1

Figure 5-A

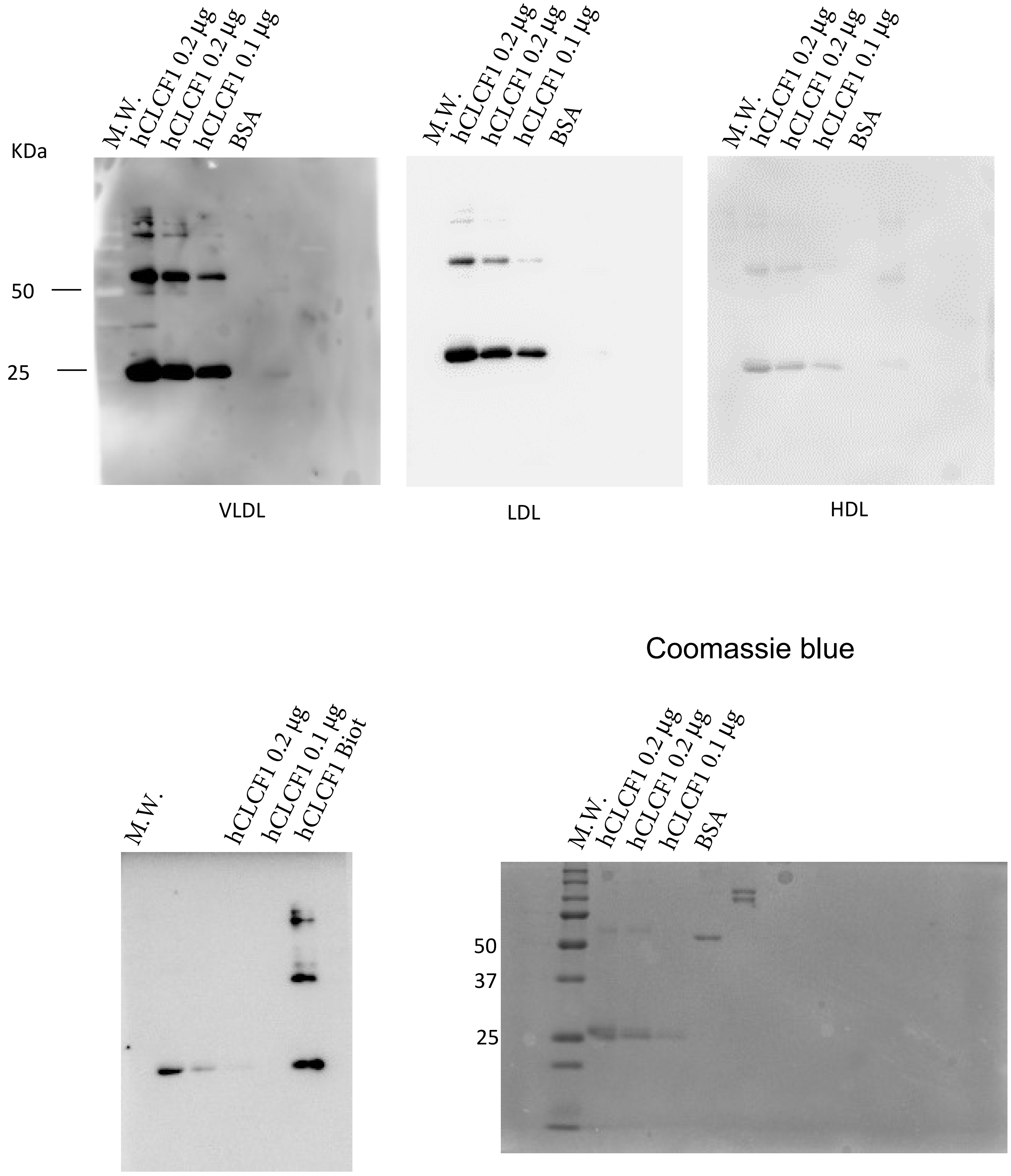

M.W.
hCLCF1 0.2 µg
hCLCF1 0.2 µg
hCLCF1 0.1 µg
BSA
KDa
50
25
VLDL
LDL
HDL
Coomassie blue
M.W.
hCLCF1 0.2 µg
hCLCF1 0.1 µg
hCLCF1 Biot
M.W.
hCLCF1 0.2 µg
hCLCF1 0.2 µg
hCLCF1 0.1 µg
BSA
50
37
25

Figure 7-A

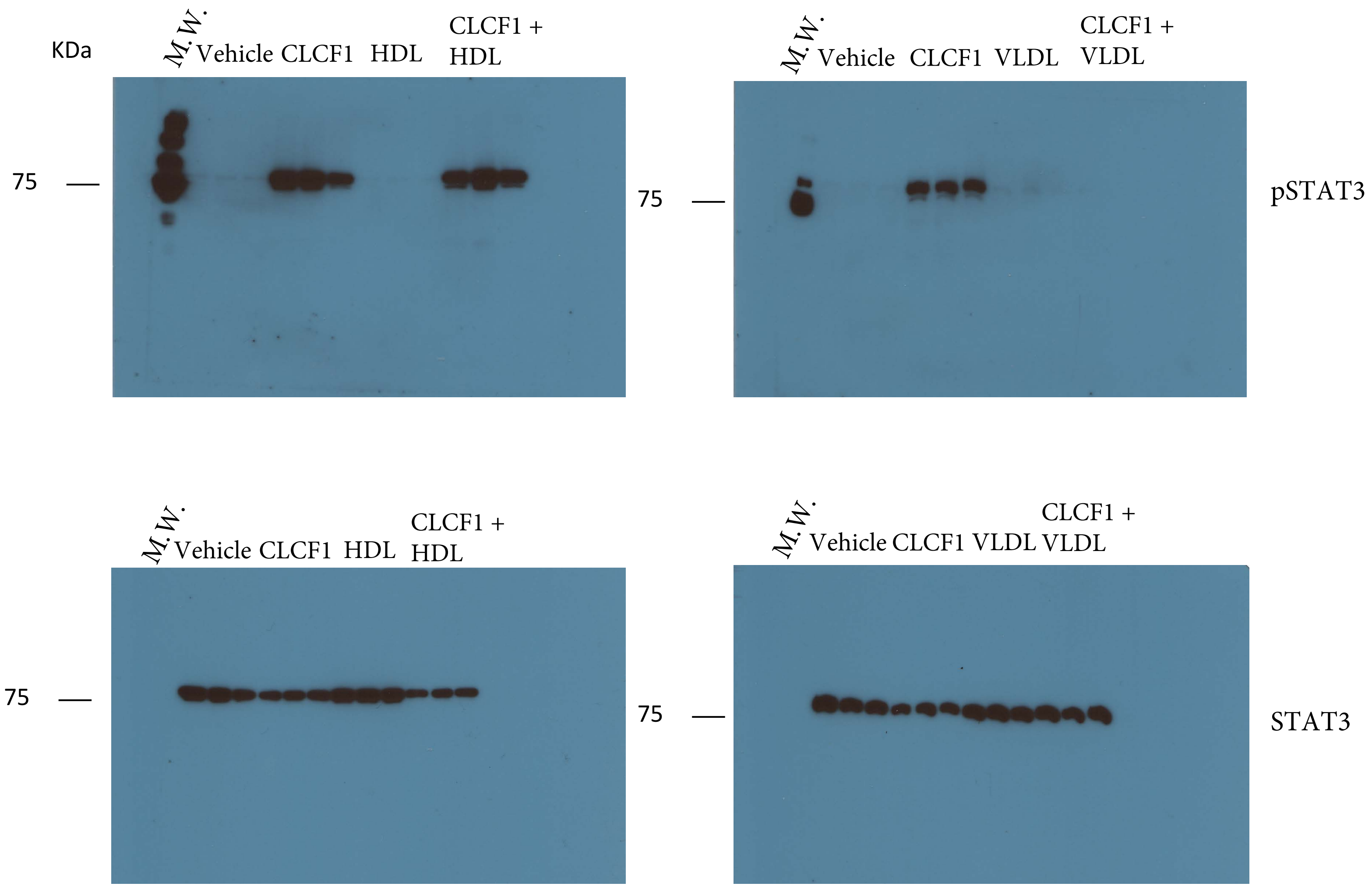
KDa
M.W.
Vehicle CLCF1 HDL
CLCF1 +
HDL
75
M.W.
Vehicle CLCF1 VLDL
CLCF1 +
VLDL
75
pSTAT3
M.W.
Vehicle CLCF1 HDL
CLCF1 +
HDL
75
M.W.
Vehicle CLCF1 VLDL
CLCF1 +
VLDL
75
STAT3
